# Discovery of Potential, Dual-Active Histamine H_3_ Receptor Ligands with Combined Antioxidant Properties

**DOI:** 10.3390/molecules26082300

**Published:** 2021-04-15

**Authors:** Kamil J. Kuder, Magdalena Kotańska, Katarzyna Szczepańska, Kamil Mika, David Reiner-Link, Holger Stark, Katarzyna Kieć-Kononowicz

**Affiliations:** 1 Department of Technology and Biotechnology of Drugs, Faculty of Pharmacy, Jagiellonian University Medical College, Medyczna 9, 30-699 Kraków, Poland; k.szczep@if-pan.krakow.pl (K.S.); mfkonono@cyf-kr.edu.pl (K.K.-K.); 2 Department of Pharmacodynamics, Faculty of Pharmacy, Jagiellonian University Medical College, Medyczna 9, 30-699 Kraków, Poland; magda.dudek@uj.edu.pl (M.K.); kamil.mika@doctoral.uj.edu.pl (K.M.); 3 Institute of Pharmaceutical and Medicinal Chemistry, Heinrich Heine University Düsseldorf, Universitaetsstr. 1, 40225 Duesseldorf, Germany; david.reiner@sund.ku.dk (D.R.-L.); stark@hhu.de (H.S.)

**Keywords:** histamine H_3_ receptor, histamine H_3_ receptor ligands, piperazine derivatives, molecular modeling, antioxidative agents

## Abstract

In an attempt to find new dual acting histamine H_3_ receptor (H_3_R) ligands, we designed a series of compounds, structurally based on previously described in our group, a highly active and selective human histamine H_3_ receptor (hH_3_R) ligand KSK63. As a result, 15 obtained compounds show moderate hH_3_R affinity, the best being the compound **17** (hH_3_R *K*_i_ = 518 nM). Docking to the histamine H_3_R homology model revealed two possible binding modes, with key interactions retained in both cases. In an attempt to find possible dual acting ligands, selected compounds were tested for antioxidant properties. Compound **16** (hH_3_R *K*_i_ = 592 nM) showed the strongest antioxidant properties at the concentration of 10^−4^ mol/L. It significantly reduced the amount of free radicals presenting 50–60% of ascorbic acid activity in the 2,2-diphenyl-1-picrylhydrazyl (DPPH) assay, as well as showed antioxidative properties in the ferric reducing antioxidant power (FRAP) assay. Despite the yet unknown antioxidation mechanism and moderate hH_3_R affinity, **16** (QD13) constitutes a starting point for the search of potential dual acting H_3_R ligands-promising tools for the treatment of neurological disorders associated with increased neuronal oxidative stress.

## 1. Introduction

**Figure 1:**
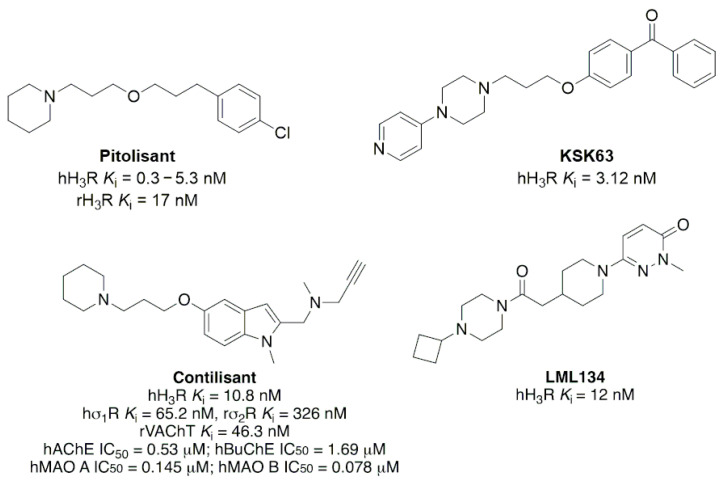
Structure and properties of reference histamine H_3_ receptor ligands [9,24,25,26] (*K*_i_-inhibitory constant, IC_50_-half maximal inhibitory concentration).

Histamine H_3_ receptor (H_3_R) is an outstanding member of the histamine receptor family. Since its discovery and gene cloning, it has served as a widely explored drug target due to its broad spectrum of neuromodulatory effects in the central nervous system (CNS) [1]. On the one hand, activation of presynaptic receptors on histaminergic neurons results in regulation of synthesis and release of the natural ligand histamine. On the other hand, activation of H_3_R as a heteroreceptor influences the whole range of other neurotransmitters, including acetylcholine, dopamine, serotonin, norepinephrine, γ-aminobutyric acid, glutamate, and substance P, to name a few [2,3,4]. Anatomically speaking, H_3_Rs are prominently expressed in humans in the basal ganglia, globus pallidus, hippocampus, and cortex–regions associated with cognition, sleep, wakefulness, and homeostatic regulation [5]. Consequently, H_3_R ligands, either antagonist or inverse agonists, as they also demonstrate constitutive activity, contribute to one of the largest groups of contemporary GPCR ligand research, with implications to treat a number of CNS-based diseases, including Alzheimer’s (AD) and Parkinson’s disease (PD), schizophrenia, ADHD, obesity, and narcolepsy [6,7]. However, while several big pharma companies were working on development of H_3_R inhibiting ligand and reached the Phase III studies in these indications, all of them failed. The only compound, pitolisant, reached the market under the name of Wakix^®^ (Figure 1), with an indication for narcolepsy treatment [8]. One exception worth mentioning is the LML134 compound (Figure 1), which completed the Phase I study by means of safety and tolerability, and went for further trials, with promising results for the treatment of excessive sleep disorders. However, the studies were terminated due to business issues [9,10].

At the same time, increasing evidence indicates that oxidative stress plays an important role in the pathogenesis of many diseases. H_3_ receptor-induced activation of G_i/o_-proteins seems to stimulate phospholipase A_2_ (PLA_2_) to induce the release of arachidonic acid, which, under pathological conditions, act as precursors for various inflammatory compounds, which in turn are crucial in mediating the oxidative and inflammatory responses in CNS pathologies, such as stroke, AD, PD and multiple sclerosis [11]. In fact, histamine H_3_ receptor ligands that might inhibit oxidative stress by reducing free radical concentrations have been shown to have potential therapeutic applications in several neurological disorders, such as Parkinson’s [12], Alzheimer’s disease [13], cerebral ischemia/stroke [14], depressive illness [15], epilepsy [16,17], schizophrenia [18], autism [19], and cardiovascular disorders, such as myocardial infarction [20]. All of these studies show that the antioxidant activities of histamine H_3_ receptor ligands play a significant role in reducing oxidative stress that accompany the above mentioned diseases.

Throughout the years, an abundant number of H_3_R ligands have been synthesized, based on various heterocyclic moieties, starting from histamine’s native imidazole moiety to (substituted) piperidines, pyrrolidines, and piperazines, among others. Yet, the overall structure remained unchanged: basic cores connected via (rigidified) alkyl chain with polar moiety, which in turn is connected, either directly or via a second linker, with a so-called “eastern arbitrary region” [21]. While the latter ones are believed to modulate potency and pharmacokinetic properties of H_3_R antagonists/inverse agonists, the heterocyclic moiety maintains the necessary interactions with binding pocket amino acids, essential for the receptor binding.

Recent publications from our group [22,23,24] allowed for the establishment of unique heterocyclic moiety, namely 4-pyridylpiperazine as a new bioisosteric replacement of piperidine. Molecular modeling studies confirmed its ability to form additional interactions, besides those of piperazine protonated nitrogen, with highly conserved H_3_R amino acids. These findings were tested in vitro, proving this scaffold to be a crucial element for high H_3_R affinity. In turn, bulky substituents of the so-called “eastern region”, such as benzophenones, have also shown to act as possible additional H-bond acceptor moiety, strengthening ligand-receptor interactions. It was again proved in vitro, resulting in one of the highest affine H_3_R ligands from our group, KSK63 (hH_3_R *K*_i_ = 3.12 nM, Figure 1).

**Figure 2:**
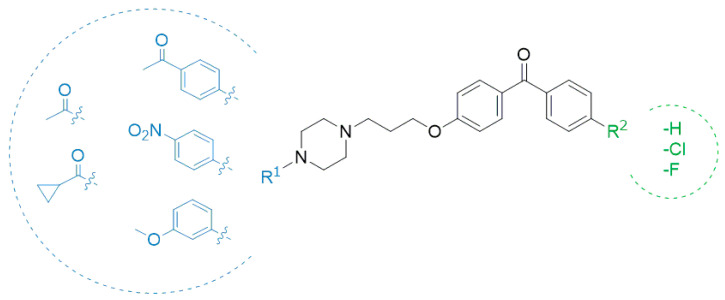
General structure of designed ligands.

In light of the presented findings, we designed the series of ligands with modifications in the hydrophilic and distal regulatory regions, namely by introducing different substituents in the piperazine (R^1^) and benzophenone (R^2^) moieties, respectively, based on the general structure depicted in Figure 2. The high activity associated with the basic part of the molecule, prompts to look for new scaffolds, even if many attempts lead to inactive compounds. For this series, we have chosen five different (cyclo)alkyl carbonyl and 4-arylpiperazine substituents. Using direct (cyclo)alkyl carbonyl piperazine *N4* nitrogen substituents, we wanted to test whether such reduction in piperazine *N4* nitrogen negative charge would still be acceptable for the retention of H_3_R affinity [27]. On the other hand, in order to avoid the possible overlap of inductive and mesomeric effects of *p*-substituted methoxy group, we have chosen *m*-substituted derivatives, so as to take only the former one into account. On the eastern end, we tested whether the increase of the hydrophobicity, by introduction of halogen atoms to benzophenone moiety, is also tolerated.

Hence, it is relevant to search for novel bioactive compounds able to combine in one molecule multiple action properties. The search for possible dual active H_3_R ligands, which might act as neuroprotectives, both directly (by expressing anti-oxidative properties) and indirectly (through the effect of H_3_R antagonism), seems necessary, as their applications might be beneficial in future therapies. Even though the obtained compounds lack the phenolic protons believed to be essential for the antioxidant activity, paying attention to, e.g., the structure of the known antioxidant melatonin [28], with respect to recent findings on the tetratargeting Contilisant [26,29], selected compounds were tested for possible antioxidative properties. Last, but not least, biological test results were complemented by the docking studies to the histamine H_3_ receptor homology model.

## 2. Results and Discussion

### 2.1. Chemistry

**Scheme 1:**
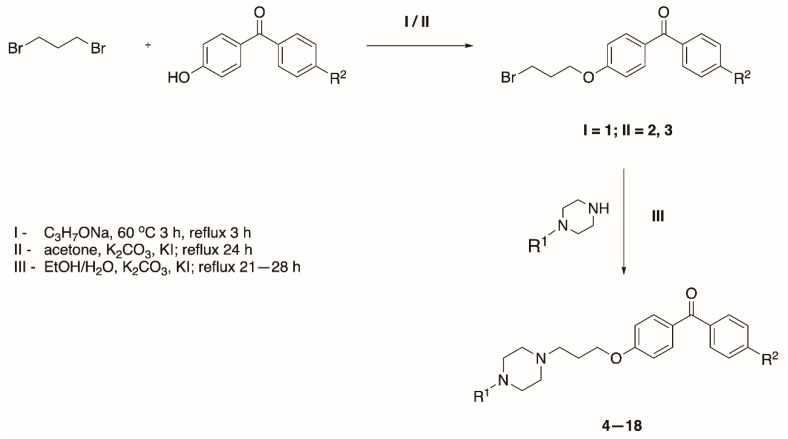
General synthetic pathway for compounds **4**–**18**.

The desired final compounds **4–18** were obtained through the synthetic route presented in Scheme 1, according to the previously reported methods [23,30]. Starting phenoxy-alkyl bromides were obtained in one-step O-alkylation of commercially available (substituted) p-hydroxy benzophenones with 1,3-dibromopropane, refluxed either in sodium propanolate (**1**) or acetone with powdered potassium carbonate, and a catalytic amount of potassium iodide (**2**, **3**). Obtained precursor bromides were then coupled with commercially available 4-substituted piperazinyl derivatives in the mixture of ethanol/water with powdered potassium carbonate and a catalytic amount of potassium iodide. Final products were obtained either as quick crystallizing free bases (**5**, **10**–**15**), or were isolated as oxalic acid salts (**4**, **6**–**9**, **16**–**18**). For detailed information, please refer to the Materials and Methods section.

### 2.2. Biological Evaluation

All compounds, in either basic or oxalate forms, were then tested in H_3_R binding studies in vitro, as described previously [31,32]. In brief, compounds were tested at five to eleven appropriate concentrations in a [^3^H]*N*^α^-methylhistamine (*K*_D_ = 3.08 nM) radioligand depletion assay to determine the affinity for the human recombinant histamine H_3_R stably expressed in HEK-293 cells. For poorly soluble compounds, **16** and **17**, an addition to 0.025% HCl in the binding test buffer was used.

Human H_3_R binding characterization of the tested compounds are presented in Table 1. Most of the tested compounds show relatively low to no histamine H_3_ receptor affinity, with the exception of *p*-nitro- (**14**, **15**) and *m*-methoxyphenyl derivatives (**16**–**18**), which display affinities at moderate levels, with compound **17** being the most affine (hH_3_R *K*_i_ = 518 nM) of the group. Regarding the piperazine substituents, one could observe that direct substitution of piperazine *N*4 nitrogen with simple (cyclo)alkyl carbonyl moieties has negative influence on hH_3_R affinity. Slightly higher values were demonstrated for *p*-acetylphenyl derivatives; however, still at an unacceptable level. It appears that such a group is not suitable for histamine H_3_ receptor binding. The reasoning behind these observations might be the low pK_a_ value of *N*4 nitrogen of the obtained compounds. With, e.g., pK_a_ = −6.0 for compounds **4**–**6,** it might be assumed that the majority of the compounds at physiological pH are in non-ionized form. This might result in a lack of crucial interactions with binding pocket amino acids, resulting in very low or no affinity at all. It appears that only *m*-methoxy- (**16**–**18**) and *p*-nitrophenyl derivatives (**14**, **15**) express the pK_a_ at a level that allows for abundance of ionized forms. Calculated pK_a_ values can be found in Table S1 of the Supplementary Material.

The overall affinity row for 4-piperazinyl substituents can be summarized as *m-*methoxyphenyl > *p*-nitrophenyl > *p*-acetylphenyl > cyclopropylmethanone > acyl (best to worst) for the herein presented series. Moreover, no clear structure–affinity relationship among the three benzophenone modified derivatives can be drawn.

### 2.3. Antioxidative Properties

In order to determine the possible antioxidant properties, selected compounds were tested in 2,2-diphenyl-1-picrylhydrazyl (DPPH) and ferric reducing antioxidant power (FRAP) assay with ascorbic acid as a reference compound (Table 2). For details, please refer to the Materials and Methods Section. Graphical representation of tests results can be found in Supplementary Material.

Antioxidant activity at the level above 50% of ascorbic acid activity at a concentration of 10^−4^ mol/L might be considered noteworthy. In the DPPH assay, compound **16** (QD13) at a concentration of 10^−4^ mol/L caused a decrease in absorbance by 12.37 ± 1.31% when compared to the sample containing the solvent + reaction mixture (blank, maximum concentration of the DPPH radical). Moreover, **16,** at a concentration of 10^−4^, had 60.28% of the ascorbic acid activity (10^−4^ mol/L, calculated from Table 2). In fact, of all the tested compounds, **16** expressed the strongest antioxidant properties.

Eight of the other compounds showed a slightly smaller but pronounced decrease in absorbance (antioxidant activity > 20% of ascorbic acid activity at 10^−4^ mol/L); thus, scavenging DPPH free radicals (Table 2, left panel).

On the other hand, in the FRAP assay, compounds **16**–**18** showed highest activity of all the tested compounds. The level of absorbance at a concentration 10^−4^ mol/L was above 8% of ascorbic acid activity at a concentration of 10^−4^ mol/L (Table 2, right panel).

Considering both tests results, compound **16** showed the strongest antioxidant properties. At a concentration of 10^−4^ mol/L, it significantly reduced the number of free radicals (DPPH test) and exhibited antioxidative properties in general (FRAP test) despite yet unknown mechanism. Furthermore, other compounds from the *m*-methoxyphenyl subgroup, **17** and **18**, also showed antioxidant activity in FRAP assay. Among three various phenyl (Ph) substituents, only methoxy derivatives appeared to possess antioxidant properties. This might be due to the fact that the *p*-NO_2_–Ph, *p*-COCH_3_–Ph are capable of attracting electrons, instead of giving them, while *p*-OCH_3_–Ph are proficient in giving electrons rather than capturing them, which in turn is an indication of their antioxidant activity. However, with such an electron withdrawing substituent as *m*-OCH_3_, antioxidant activity appears in the studied group of compounds.

Overall, these results prompt for future investigation of compounds **16**–**18** antioxidant properties in various disease entities. Despite its moderate affinity for histamine H_3_ receptors, **16** (QD13) could be investigated as an adjuvant to known therapies in many diseases, to which contributes an oxidative stress and where modulation of the brain levels of histamine and other neurotransmitters provide improvement.

### 2.4. Docking Studies

Three out of four known and described histamine receptors still remain a Gordian knot by means of crystallography. To date, only histamine H_1_ receptor crystal structure was resolved (PDB ID: 3RZE [33]). Therefore, we used a previously described histamine H_3_R homology model in our study, which was constructed on the template of crystal structure of M_2_ muscarinic acetylcholine receptor (PDB ID: 3UON) [22]. Considering that, due to very low basicity, the abundance of compounds at physiological pH might appear in non-ionized forms, for docking studies, we prepared both protonated and non-protonated conformers.

All of the compounds were docked and, independently of the form, fit the H_3_R binding pocket. In case of non-protonated conformers, compounds were placed shallower in the binding pocket, and mostly stabilized through π–π stacking or additional H-bonds with Y374 (3.51) or R381 (6.58). However, no interactions with either of the key amino acids, E206 (5.46) and D114 (3.32), were found. For possible non-ionized forms, this might explain very low or no affinity for the histamine H_3_ receptor. Calculated binding poses for non-protonated conformers can be found in the Supplementary Material (Table S2).

**Figure 3:**
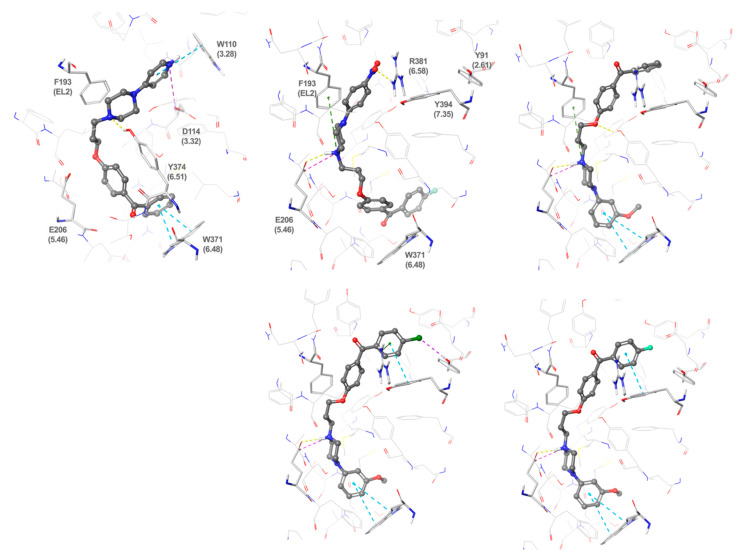
Putative binding poses of protonated conformers. Upper panel: KSK63 (left) **15** (middle) and **16** (right); lower panel: **17** (middle), **18** (right) in histamine H3 homology receptor.

Nonetheless, two different binding modes were surprisingly observed for protonated conformers: “standard (ST)”, with the eastern region pointing to the extracellular space, and “upside-down (UD)” (Figure 3). The positions of protonated basic moieties of the ligands were mostly similar, independently of the mode. In both cases, a crucial histamine H_3_R antagonist/inverse agonist interaction-salt bridge and/or hydrogen bond formation between protonated amine nitrogen and E206 (5.46) was, however, preserved due to the approximate location of the protonated nitrogen (superscripts denote Ballesteros–Weinstein numbering [34]).

In the ST mode, benzophenone fragments occupied the space fenced by aromatic features of F193, Y189 (ECL2), and Y394 (7.35) on the sides, and Y91 (2.61) and Y94 (2.64) on the top. In most cases, a hydrogen bond between the carbonyl group and R381(6.58) was found. The latter one was not observed for inactive compounds **4**–**9**. Additional stabilization through halogen bond formation with Y91 (2.61) and π–π stacking with Y394 (7.35) and W371 (6.48), in the case most active for this series of *m*-methoxyphenyl derivatives, was also present. These interactions might explain the overall higher H_3_R affinity of the ligands from this subgroup.

On the other hand, the UD mode was observed for *p*-acetylphenyl and *p*-nitrophenyl derivatives, with the exception of **18**, for which both modes were observed. Although, the ST mode expressed slightly lower glide energy (−58.78 vs −56.08 kcal/mol). In this mode, the benzophenone fragments reached deeper towards the narrow sub pocket localized among the transmembrane regions (TM) 1, TM3, and TM7. The latter is formed by lateral L117 (3.35) and W371 (6.48), and S121 (3.39) and A122 (3.40) from the bottom. Additional pose stabilization through cation–π interactions between protonated piperazine nitrogen and F193 (ECL2), as well as the hydrogen bond between the carbonyl/nitro group and R381 (6.58) was found.

Along with the novel compounds, reference structure KSK63 was also docked. In this case the pyridyl moiety nitrogen is protonated instead of the piperazine one. This allows for a shift of the ligand towards TM3 and formation of ionic interaction with key aminergic GPCR anchor, D114 (3.32) (Figure 3). Lack of such interactions observed for the novel, described compounds, might also be responsible for their much lower affinity towards histamine H_3_ receptor.

**Figure 4:**
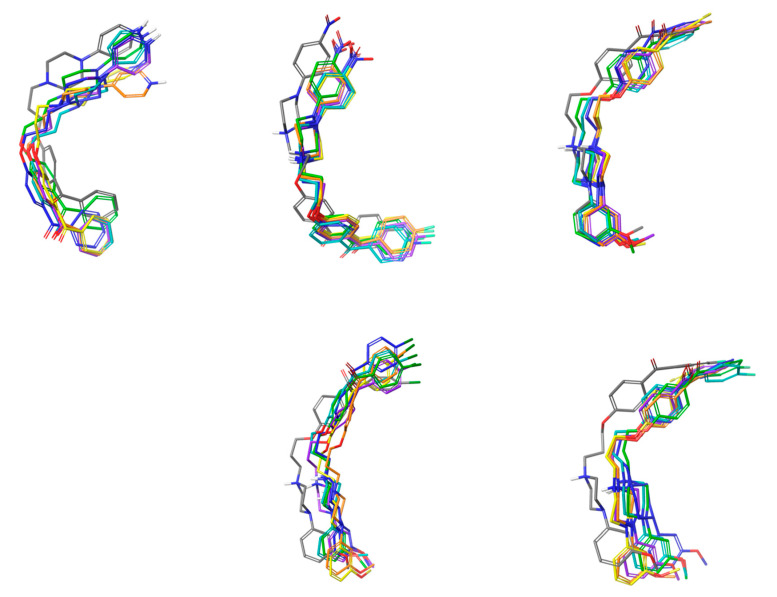
Orientations of: upper panel-KSK63 (left), **15** (middle), and **16** (right); lower panel-**17** (middle), 18 (right) during the molecular dynamics simulations in 0ps–grey, 100ps–green, 200ps–teal, 300ps–green, 400ps–violet, 500ps–orange, 600ps–yellow.

The stability of the calculated poses for this groups’ most affine compounds **15**–**17** and both orientations of **18** along with the reference compound KSK63 was further evaluated by means of molecular dynamics (MD) simulations. From each simulation, 7 poses were selected (starting pose, and after each 100ps up to 600ps). In the case of **15**–**17** (Figure 4) and KSK63, complexes appeared stable through the whole 600ps simulation, retaining the key interactions, and the potential energy (U) of the atomic system at the level of ~1000 kcal/mol.

**Figure 5:**
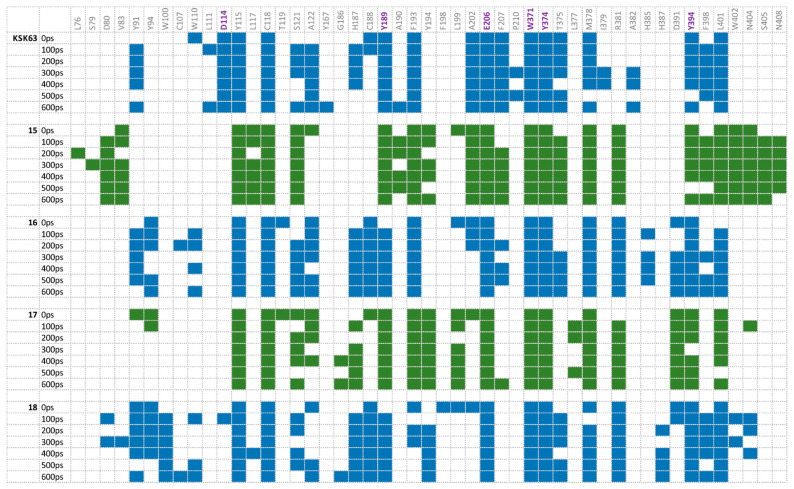
Interaction fingerprints of compounds KSK63, **15**–**18** with H_3_R model during molecular dynamics (MD) simulation for particular frames. Colored boxes indicate any interaction between the ligand and corresponding amino acid. Key interaction amino acids were marked violet bold.

In all cases, starting poses (marked in grey, Figure 4) were very similar to its orientation at the end of simulation (marked yellow). However, a shift in the binding site with retained conformation appeared in the first 100ps of the simulation. On the other hand, the analysis of KSK63 behavior allowed for possible explanation of its’ high affinity. The conformation remained quite stable during the simulation with consistent set of interactions. What was not observed for the remaining structures, the protonated pyridyl nitrogen retained the interactions with not only the key ionic anchor D114 (3.32) but also with E206 (5.46)_,_ through the whole simulation. Detailed analysis of compounds behavior during MD simulations is provided by examining the changes in their interactions with H_3_R (Figure 5). The results indicate a relatively consistent set of ligand-protein interactions occurring during the whole MD simulation. Most of the interactions occur within the TM3, TM5, TM6 and TM7 and ECL2, with consistent interactions with Y115 (3.33), Y189 (45.51), E206 (5.46), Y394 (7.35). In the case of **18**, the standard orientation seems more stable during the simulation, with most of the starting key interactions retained in the last frame.

## 3. Materials and Methods

### 3.1. Chemistry

All reagents were purchased from commercial suppliers (Alfa Aesar, Sigma-Aldrich, CHESS GmbH, Chempur) and were used without further purification. Melting points (mp) were determined on a Büchi Melting Point M560 apparatus (BÜCHI Labortechnik AG, Flawil, Switzerland) and were uncorrected. Liquid chromatography/mass spectra (LC/MS) were obtained using Waters TQ Detector mass spectrometer (Milford, MA, USA). ^1^H NMR spectra were recorded on a Varian Mercury 300 MHz PFG (Palo Alto, CA, USA) spectrometer in DMSO-*d*_6_. Chemical shifts were expressed in parts per million (ppm) using the solvent signal as an internal standard. Data have been reported in the following order: multiplicity (s, singlet; d, doublet; t, triplet; qi, quintet; m, multiplet; br, broad; Ac, acetyl; Prop, propionyl; t–b, tert–butyl; t–p, tert–pentyl; Ph, phenyl; Pip, piperazine), approximate coupling constants *J* expressed in Hertz (Hz), number of protons. ^13^C NMR spectra were recorded on Varian-Mercury-VX 300 MHz PFG or Bruker 400 MHz spectrometer (Berlin, Germany) at 75 MHz in DMSO-*d*_6_. LC–MS separation was carried out on a system consisting of a Waters ACQUITY UPLC, coupled to a Waters TQD mass spectrometer. Retention times (t_R_) were stated in minutes. For flash chromatography (FC) purification, silica gel 60 (0.063–0.20 mm; Merck) and the following solvent systems were used: I-petroleum ether:EtOAc (97.5:1.25); II-CH_2_Cl_2_:MeOH (95:5).

#### 3.1.1. Procedure 1

To a solution of proper hydroxy benzophenone (0.01 mol) in acetone (60 mL), anhydrous potassium carbonate (1.12 g, 0.008 mol) and catalytic amount of potassium iodide were added. The reaction mixture was stirred for 10 min at room temperature, after which 1,3-dibromopropane (12 g, 0.06 mol) was added. The reaction mixture was then heated at reflux for 24 h, after which the inorganic solids were filtered, and acetone was evaporated. Remaining oils were then purified by flash chromatography (system I).

*4-(3-Bromopropoxyphenyl)(phenyl)methanone* (**1**). Synthesis from (4-hydroxyphenyl) (phenyl)methanone (1.98 g, 0.01 mol). Obtained 2.78 g of white quickly crystalizing oil. C_16_H_15_BrO_2_, Molecular Weight (MW) = 319.20, Yield = 87%.

*4-(3-Bromopropoxyphenyl)(4-chlorophenyl)methanone* (**2**). Synthesis from (4-hydroxyphenyl)(4-chlorophenyl)methanone (2.32 g, 0.01 mol). Obtained 2.13 g of white quickly crystalizing oil. C_16_H_14_BrClO_2_, MW = 351.99, Yield = 60%)

#### 3.1.2. Procedure 2

Procedure was performed according to the literature reference [30]. To a solution of freshly prepared sodium propanolate (25 mL propanol; 0.575 g, 0.025 mol Na), (4-fluorophenyl) (4-hydroxyphenyl)methanone (5.4 g, 0.025 mol) was added and stirred at room temperature (RT) for 15 min; 1,3-dibromopropane (15.14 g, 0.075 mol) was then added dropwise over one hour. The reaction mixture was stirred at 60 °C for 3 h, and then refluxed for another 3 h. After cooling down to RT reaction mixture was filtrated and evaporated. To a resulting brown oil, MeOH was added, and purified by flash chromatography (system II); obtaining 6 g of yellowish, quickly crystallizing oil.

*4-(3-Bromopropoxyphenyl)(4-fluorophenyl)methanone* (**3**). C_16_H_14_BrFO_2_, MW = 337.18, yield = 71%

#### 3.1.3. Procedure 3

Final products were obtained according to the procedure described in [22] and purified by flash chromatography (CH_2_Cl_2_/MeOH, 95:5). Resulting oils were either: transformed into oxalic acid salts, using 10% excess of oxalic acid solution in absolute ethanol in RT, and then precipitated by addition of ethyl ether or, in the case of self-crystalizing oils, remained free bases.

*1-(4-(3-(4-Benzoylphenoxy)propyl)piperazin-1-yl)ethan-1-one hydrogen oxalate* (**4**, QD11). Synthesis from **1** (0.47 g, 1.5 mmol) and 1-(piperazin-1-yl)ethan-1-one (0.47 g, 2.5 mmol). Purified by FC. Obtained 550 mg of white oil, transferred into oxalic acid salt. Yield = 63%; mp = 136.8–140 °C; UPLC/MS purity 98.20%, t_R_ = 5.05, C_22_H_26_N_2_O_3_ × C_2_H_2_O_4_, MW = 366.46, [M + H]^+^ 367.25. ^1^H NMR (500 MHz, DMSO-*d*_6_) δ ppm: 7.69–7.72 (m, 2H, Ph), 7.60–7.66 (m, 3H, Ph), 7.49–7.54 (m, 2H, Ph), 7.03–7.07 (m, 2H Ph), 4.11 (t, *J* = 6.01 Hz, 2H, CH_2_-O), 3.47–3.57 (m, 4H, Pip-2,6H), 2.75–2.83 (m, 4H, Pip-3,5H), 2.68–2.74 (m, 2H, NCH_2_), 1.98–2.04 (m, 2H, CH_2_), 1.97 (s, 3H, COCH_3_). ^13^C NMR (DMSO-d6): 194.96, 168.86, 164.29, 162.71, 138.29, 132.74, 129.78, 129.00, 114.89, 66.32, 54.13, 52.56, 52.14, 44.73, 25.18, 21.62.

*1-(4-(3-(4-(4-Chlorobenzoyl)phenoxy)propyl)piperazin-1-yl)ethan-1-one* (**5**, QD15). Synthesis from **2** (0.53 g, 1.5 mmol) and 1-(piperazin-1-yl)ethan-1-one (0.19 g, 1.5 mmol). Purified by FC. Obtained 330 mg of light yellow free-base solid. Yield = 37%; mp = 123.9–126.7 °C; UPLC/MS purity 100%, t_R_ = 4.63, C_22_H_25_ClN_2_O_3_, MW = 400.90, [M + H]^+^ 401.14. ^1^H NMR (500 MHz, DMSO-*d*_6_) δ ppm: 7.65–7.72 (m, 4H, Ph), 7.57 (d, *J* = 8.02 Hz, 2H, Ph), 7.04 (d, *J* = 8.59 Hz, 2H, Ph), 4.09 (t, *J*=6.30 Hz, 2H, CH_2_-O), 3.34–3.41 (m, 4H, Pip-2,6H), 2.41 (t, *J* = 7.16 Hz, 2H, NCH_2_), 2.31–2.36 (m, 2H, Pip-3,5H), 2.27 (t, *J* = 4.87 Hz, 2H, Pip-3,5H), 1.94 (s, 3H, COCH_3_), 1.88 (quin, *J* = 6.73 Hz, 2H, CH_2_). ^13^C NMR (DMSO-*d*_6_): 193.80, 168.60, 163.08, 137.48, 137.00, 132.76, 131.68, 129.12, 114.94, 66.80, 54.68, 53.55, 53.02, 46.18, 41.36, 26.48, 21.71.

*1-(4-(3-(4-(4-Fluorobenzoyl)phenoxy)propyl)piperazin-1-yl)ethan-1-one hydrogen oxalate* (**6**, QD4). Synthesis from **3** (0.50 g, 1.5 mmol) and 1-(piperazin-1-yl)ethan-1-one (0.32 g, 2.5 mmol). Purified by FC. Obtained 620 mg of yellow-green oil, transferred into oxalic acid salt. Yield = 70%; mp = 171.3–173.1 °C; UPLC/MS purity 100%, t_R_ = 4.26, C_22_H_25_FN_2_O_3_ × C_2_H_2_O_4_, MW = 384.45 (+90.04), [M + H]^+^ 385.19. ^1^H NMR (500 MHz, DMSO-*d*_6_) δ ppm: 7.69–7.75 (m, 4H, Ph), 7.32–7.37 (m, 2H, Ph), 7.04–7.07 (m, 2H, Ph), 4.12 (t, *J* = 6.01 Hz, 2H, CH_2_-O), 3.57–3.66 (m, 4H, Pip-3,5H), 3.01–3.07 (m, 4H, Pip-2,6H), 2.97 (br. s., 2H, NCH_2_), 2.05–2.14 (m, 2H, CH_2_), 2.00 (s, 3H, COCH_3_). ^13^C NMR (DMSO-*d*_6_): 193.63, 169.01, 163.88, 163.68, 162.59, 134.76, 132.68, 129.95, 116.16, 115.98, 114.93, 66.06, 53.77, 51.95, 51.59, 43.83, 24.37, 21.56.

*(4-(3-(4-Benzoylphenoxy)propyl)piperazin-1-yl)(cyclopropyl)methanone hydrogen oxalate* (**7**, QD14)**.** Synthesis from **1** (0.47 g, 1.5 mmol) and cyclopropyl(piperazin-1-yl)methanone (0.23 g, 1.5 mmol). Purified by FC. Obtained 520 mg of white oil, transferred into oxalic acid salt. Yield = 63%; mp = 167.3–168.6 °C; UPLC/MS purity 100%, t_R_ = 4.30, C_24_H_28_N_2_O_3_ × C_2_H_2_O_4_, MW = 392.21, [M + H]^+^ 393.41. ^1^H NMR (500 MHz, DMSO-*d*_6_) δ ppm: 7.69–7.74 (m, 2H, Ph), 7.59–7.66 (m, 3H, Ph), 7.49–7.54 (m, 2H, Ph), 7.03–7.08 (m, 2H, Ph), 4.12 (t, *J* = 6.30 Hz, 2H, CH_2_-O), 3.84 (br. s., 2H, Pip-3,5H), 3.60 (br. s., 2H, Pip-3,5H), 2.92–3.03 (m, 6H, Pip-2,6H + NCH_2_), 2.03–2.11 (m, 2H, CH_2_), 1.93–1.99 (m, 1H, Cp), 0.66–0.74 (m, 4H, Cp). ^13^C NMR (DMSO-*d*_6_): 194.96, 171.76, 164.19, 162.63, 138.28, 132.73, 129.78, 129.00, 114.90, 66.18, 53.92, 24.69, 10.73, 7.70

*(4-(3-(4-(4-Chlorobenzoyl)phenoxy)propyl)piperazin-1-yl)(cyclopropyl)methanone hydrogen oxalate* (**8**, QD19)**.** Synthesis from **2** (0.28 g, 0.78 mmol) and cyclopropyl(piperazin-1-yl)methanone (0.12 g, 0.78 mmol). Purified by FC. Obtained 220 mg of light-yellow oil solid, transferred into oxalic acid salt. Yield = 26%; mp = 176.9–179.2 °C; UPLC/MS purity 100%, t_R_ = 4.99, C_24_H_27_ClN_2_O_3_, MW = 426.94, [M + H]^+^ 427.41. ^1^H NMR (500 MHz, DMSO-*d*_6_) δ ppm: 7.69–7.73 (m, 2H, Ph), 7.64–7.69 (m, 2H, Ph), 7.56–7.60 (m, 2H, Ph), 7.03–7.08 (m, 2H, Ph), 4.12 (t, *J* = 6.01 Hz, 2H, CH_2_-O), 3.83 (br. s., 2H, Pip-3,5H), 3.60 (br. s., 2H, Pip-3,5H), 2.91–3.03 (m, 4H, Pip-2,6H), 2.88 (br. s., 2H, NCH_2_), 2.02–2.10 (m, 2H, CH_2_), 1.92–2.00 (m, 1H, Cp), 0.65–0.73 (m, 4H, Cp).). ^13^C NMR (DMSO-*d*_6_): 193.83, 171.75, 164.12, 162.79, 137.52, 136.95, 132.75, 131.69, 129.13, 114.98, 66.22, 53.91, 24.71, 10.73, 7.70

*(4-(3-(4-(Cyclopropanecarbonyl)piperazin-1-yl)propoxy)phenyl)(4-fluorophenyl)methanone hydrogen oxalate* (**9**, QD8). Synthesis from **3** (0.50 g, 1.5 mmol) and cyclopropyl(piperazin-1-yl)methanone (0.23 g, 1.5 mmol). Purified by FC. Obtained 530 mg of light oil, transferred into oxalic acid salt. Yield = 64%; mp = 181.1–182.7 °C; UPLC/MS purity 100%, t_R_ = 4.48, C_24_H_27_FN_2_O_3_ × C_2_H_2_O_4_, MW = 410.48 (+90.04), [M + H]^+^ 411.18. ^1^H NMR (500 MHz, DMSO-*d*_6_) δ ppm: 7.68–7.76 (m, 4H, Ph), 7.32–7.37 (m, 2H, Ph), 7.03–7.07 (m, 2H, Ph), 4.12 (t, *J* = 6.01 Hz, 2H, CH_2_-O), 3.83 (br. s., 2H, Pip-2,6H), 3.59 (br. s., 2H, Pip-2,6H), 2.90–3.00 (m, 4H, Pip-3,5H), 2.87 (br. s., 2H, NCH_2_), 2.03–2.11 (m, 2H, CH_2_), 1.96 (tt, *J* = 7.73, 4.87 Hz, 1H, Cp), 0.66–0.74 (m, 4H, Cp). ^13^C NMR (DMSO-*d*_6_): 193.62, 171.75, 165.87, 164.08, 162.65, 134.80, 132.69, 129.90, 116.15, 115.98, 114.93, 66.20, 53.94, 24.74, 10.72, 7.70.

*1-(4-(4-(3-(4-Benzoylphenoxy)propyl)piperazin-1-yl)phenyl)ethan-1-one* (**10**, QD10). Synthesis from **1** (0.47 g, 1.5 mmol) and 1-(4-(piperazin-1-yl)phenyl)ethan-1-one (0.51 g, 2.5 mmol). Purified by FC. Obtained 300 mg of yellow free-base solid. Yield = 62%; mp = 148.4–151.4 °C; UPLC/MS purity 100%, t_R_ = 4.94, C_28_H_30_N_2_O_3_, MW = 442.56, [M + H]^+^ 443.22. ^1^H NMR (500 MHz, DMSO-*d*_6_) δ ppm: 7.76 (d, *J* = 9.17 Hz, 2H, Ph), 7.69–7.72 (m, 2H, Ph), 7.59–7.66 (m, 3H, Ph), 7.49–7.53 (m, 2H, Ph), 7.03–7.07 (m, 2H, Ph), 6.92 (d, *J* = 9.17 Hz, 2H, Ph), 4.10 (t, *J* = 6.30 Hz, 2H, CH_2_-O), 3.26–3.31 (m, 6H, Pip-2,6H + NCH_2_), 2.45–2.47 (m, 4H, Pip-3,5H), 2.41 (s, 3H, COCH_3_), 1.91 (quin, *J* = 6.73 Hz, 2H, CH_2_). ^13^C NMR (DMSO-*d*_6_): 196.08, 194.94, 162.95, 154.38, 138.33, 132.75, 130.60, 129.77, 128.98, 114.88, 113.58, 66.81, 54.77, 52.99, 47.18, 26.62.

*1-(4-(4-(3-(4-(4-Chlorobenzoyl)phenoxy)propyl)piperazin-1-yl)phenyl)ethan-1-one* (**11**, QD17). Synthesis from **2** (0.53 g, 1.5 mmol) and 1-(4-(piperazin-1-yl)phenyl)ethan-1-one (0.3 g, 1.5 mmol). Purified by FC. Obtained 260 mg of yellowish free-base solid. Yield = 35%; mp = 180.6–183.4 °C; UPLC/MS purity 100%, t_R_ = 5.49, C_28_H_29_ClN_2_O_3_, MW = 477.00, [M + H]^+^ 477.18. ^1^H NMR (500 MHz, DMSO-*d*_6_) δ ppm: 7.76 (m, *J* = 8.88 Hz, 2H, Ph), 7.65–7.71 (m, 4H, Ph), 7.56–7.59 (m, 2H, Ph), 7.04–7.08 (m, 2H, Ph), 6.93 (d, *J* = 9.16 Hz, 2H, Ph), 4.11 (t, *J* = 6.30 Hz, 2H, CH_2_-O), 3.29–3.31 (m, 10H, Pip-3,5H + Pip-2,6H + NCH_2_), 2.41 (s, 3H, COCH_3_), 1.88–1.95 (m, 2H, CH_2_). ^13^C NMR (DMSO-*d*_6_): 196.30, 163.10, 158.53, 132.79, 131.70, 130.61, 129.45, 129.13, 114.96, 113.59, 63.87, 53.00, 26.64.

*1-(4-(4-(3-(4-(4-Fluorobenzoyl)phenoxy)propyl)piperazin-1-yl)phenyl)ethan-1-one* (**12**, QD3). Synthesis from **3** (0.50 g, 1.5 mmol) and 1-(4-(piperazin-1-yl)phenyl)ethan-1-one (0.51 g, 2.5 mmol). Purified by FC. Obtained 230 mg of yellowish free-base solid. Yield = 31%; mp = 162–163.6 °C; UPLC/MS purity 98.12%, t_R_ = 5.11, C_28_H_29_FN_2_O_3_, MW = 460.55, [M + H]^+^ 461.23. ^1^H NMR (500 MHz, DMSO-*d*_6_) δ ppm: 7.69–7.78 (m, 6H, Ph), 7.34 (t, *J* = 8.88 Hz, 2H, Ph), 7.06 (m, *J* = 9.17 Hz, 2H, Ph), 6.93 (m, *J* = 9.16 Hz, 2H, Ph), 4.11 (t, *J* = 6.30 Hz, 2H, CH_2_-O), 2.44–2.47 (m, 10H, Pip-3,5H + Pip-2,6H + NCH2), 2.41 (s, 3H, COCH_3_), 1.92 (m, *J* = 6.87 Hz, 2H, CH_2_). ^13^C NMR (DMSO-*d*_6_): 196.12, 162.97, 132.72, 130.60, 129.70, 116.14, 115.97, 114.91, 113.59, 66.83, 54.77, 53.00, 47.18, 26.63, 26.54.

*(4-(3-(4-(4-Nitrophenyl)piperazin-1-yl)propoxy)phenyl)(phenyl)methanone* (**13**, QD12). Synthesis from **1** (0.47 g, 1.5 mmol) and 1-(4-nitrophenyl)piperazine (0.31 g, 1.5 mmol). Purified by FC. Obtained 130 mg of yellow, quick crystalizing oil. Yield = 18%; mp = 182.3–183.9 °C; UPLC/MS purity 100%, t_R_ = 5.28, C_26_H_27_N_3_O_4_, MW = 445.51, [M + H]^+^ 446.40. ^1^H NMR (500 MHz, DMSO-*d*_6_) δ ppm: 8.00–8.03 (m, 2H, Ph), 7.69–7.72 (m, 2H, Ph), 7.63–7.66 (m, 2H, Ph), 7.60–7.62 (m, 1H, Ph), 7.49–7.53 (m, 2H, Ph), 7.04–7.07 (m, 2H, Ph), 7.04–7.07 (m, 2H, Ph), 4.11 (t, *J* = 6.44 Hz, 2H, CH_2_-O), 3.40–3.44 (m, 8H, Pip-3,5H + Pip-2,6H), 3.27 (m, 2H, NCH_2_), 1.92 (m, *J* = 7.02 Hz, 2H, CH_2_). ^13^C NMR (DMSO-*d*_6_): 196.19, 168.03, 164.95, 156.22, 132.77, 129.78, 129.00, 126.26, 114.89, 113.13, 66.80, 52.87, 46.84, 25.76.

*(4-Chlorophenyl)(4-(3-(4-(4-nitrophenyl)piperazin-1-yl)propoxy)phenyl)methanone* (**14**, QD20). Synthesis from **2** (0.53 g, 1.5 mmol) and 1-(4-nitrophenyl)piperazine (0.31 g, 1.5 mmol). Purified by FC. Obtained 500 mg of yellow, quick crystallizing oil. Yield = 67%; mp = 154.5–155.8 °C; UPLC/MS purity 100%, t_R_ = 5.83, C_26_H_27_ClN_3_O_4_, MW = 479.96, [M + H]^+^ 480.39. ^1^H NMR (500 MHz, DMSO-*d*_6_) δ ppm: 8.01 (d, *J* = 9.74 Hz, 2H, Ph), 7.66 (d, *J* = 8.02 Hz, 2H, Ph), 7.70 (d, *J* = 9.17 Hz, 2H, Ph), 7.57 (d, *J* = 8.59 Hz, 2H, Ph), 7.05 (d, *J* = 8.59 Hz, 2H, Ph), 6.99 (d, *J* = 9.17 Hz, 2H, Ph), 4.11 (t, *J* = 6.01 Hz, 2H, CH_2_-O), 3.41 (br. s., 4H, Pip-3,5H), 3.23–3.37 (m, 6H, Pip-2,6H + NCH_2_), 1.88–1.96 (m, 2H, CH_2_). ^13^C NMR (DMSO-*d*_6_): 193.81, 163.09, 155.28, 137.48, 137.37, 137.00, 132.77, 131.68, 129.12, 126.24, 114.95, 113.12, 66.83, 54.65, 52.86, 46,85, 26.51.

(*4-Fluorophenyl)(4-(3-(4-(4-nitrophenyl)piperazin-1-yl)propoxy)phenyl)methanone* (**15**, QD6). Synthesis from **3** (1 g, 3 mmol) and 1-(4-nitrophenyl)piperazine (0.62 g, 3 mmol). Purified by FC. Obtained 245 mg of yellow, quick crystallizing oil. Yield = 34%; mp = 173.3–174.2 °C; UPLC/MS purity 100%, t_R_ = 5.52, C_26_H_27_FN_3_O_4_, MW = 463.51, [M + H]^+^ 464.15. ^1^H NMR (500 MHz, DMSO-*d*_6_) δ ppm: 8.01 (m, *J* = 9.74 Hz, 2H, Ph), 7.67–7.76 (m, 4H, Ph), 7.34 (t, *J* = 8.88 Hz, 2H, Ph), 7.06 (m, *J* = 9.16 Hz, 2H, Ph), 6.99 (d, *J* = 9.74 Hz, 2H, Ph), 4.11 (t, *J* = 6.30 Hz, 2H, CH_2_-O), 3.39–3.44 (m, 4H, Pip-3,5H), 3.29 (s, 6H, Pip-2,6H + NCH_2_), 1.92 (quin, *J* = 6.59 Hz, 2H, CH_2_). ^13^C NMR (DMSO-*d*_6_): 193.62, 162.96, 155.28, 137.36, 132.71, 129.70, 126.25, 116.14, 115.97, 114.91, 113.12, 66.80, 54.66, 52.86, 46.85, 26.51.

(*4-(3-(4-(3-Methoxyphenyl)piperazin-1-yl)propoxy)phenyl)(phenyl)methanone hydrogen oxalate* (**16**, QD13). Synthesis from **1** (0.47 g, 1.5 mmol) and 1-(3-methoxyphenyl)piperazine (0.28 g, 1.5 mmol). Purified by FC. Obtained 650 mg of yellow oil, transferred into oxalic acid salt. Yield = 87%; mp = 143.6–146.1 °C; UPLC/MS purity 100%, t_R_ = 5.32, C_27_H_30_N_2_O_3_ × C_2_H_2_O_4_, MW = 430.55 (+ 90.04), [M + H]^+^ 431.44. ^1^H NMR (500 MHz, DMSO-*d*_6_) δ ppm: 7.70–7.73 (m, 2H, Ph), 7.60–7.66 (m, 3H, Ph), 7.50–7.54 (m, 2H, Ph), 7.05–7.12 (m, 3H, Ph), 6.53 (dd, *J* = 8.02, 2.00 Hz, 1H, Ph), 6.47 (t, *J* = 2.29 Hz, 1H, Ph), 6.38 (dd, *J* = 8.16, 1.86 Hz, 1H, Ph), 4.13 (t, J=6.01 Hz, 2H, CH_2_-O), 3.68 (s, 3H, CH_3_), 3.31 (br. s., 4H, Pip-3,5H), 3.10 (br. s., 4H, Pip-2,6H), 3.02–3.07 (m, 2H, NCH_2_), 2.08–2.14 (m, 2H, CH_2_). ^13^C NMR (DMSO-*d*_6_): 194.98, 164.54, 162.63, 160.80, 160.78, 151.87, 138.29, 132.75, 132.73, 129.80, 129.01, 114.93, 108.81, 105.45, 102.47, 66.18, 55.49, 53.79, 51.78, 46.69, 24.54.

*(4-Chlorophenyl)(4-(3-(4-(3-methoxyphenyl)piperazin-1-yl)propoxy)phenyl)methanone hydrogen oxalate* (**17**, QD18). Synthesis from **2** (0.53 g, 1.5 mmol) and 1-(3-methoxyphenyl)piperazine (0.28 g, 1.5 mmol). Purified by FC. Obtained 350 mg of yellow oil, transferred into oxalic acid salt. Yield = 46%; mp = 100.2–102.4 °C; UPLC/MS purity 100%, t_R_ = 5.95, C_27_H_29_ClN_2_O_3_, MW = 464.98, [M + H]^+^ 465.44. ^1^H NMR (500 MHz, DMSO-*d*_6_) δ ppm: 7.65–7.71 (m, 4H, Ph), 7.55–7.59 (m, 2H, Ph), 7.03–7.07 (m, 3H, Ph), 6.47 (dd, *J* = 8.02, 1.72 Hz, 1H, Ph), 6.39 (t, *J* = 2.29 Hz, 1H, Ph), 6.39 (t, *J* = 2.29 Hz, 1H, Ph), 4.10 (t, *J* = 6.30 Hz, 2H, CH_2_-O), 3.30 (s, 3H, CH_3_), 3.05–3.11 (m, 4H, Pip-3,5H), 2.42–2.47 (m, 6H, Pip-2,6H + NCH_2_), 1.90 (quin, *J* = 6.73 Hz, 2H, CH_2_). ^13^C NMR (DMSO-*d*_6_): 193.80, 163.11, 160.71, 152.94, 137.48, 137.00, 132.76, 131.68, 129.11, 114.95, 108.52, 104.58, 101.94, 66.88, 55.37, 53.29, 48.70, 26.58.

*(4-Fluorophenyl)(4-(3-(4-(3-methoxyphenyl)piperazin-1-yl)propoxy)phenyl)methanone hydrogen oxalate* (**18**, QD9). Synthesis from **3** (0.4 g, 1 mmol) and 1-(3-methoxyphenyl)piperazine (0.19 g, 1 mmol). Purified by FC. Obtained 430 mg of light oil, transferred into oxalic acid salt. Yield = 57%; mp = 127.4–129.7 °C; UPLC/MS purity 100%, t_R_ = 5.47, C_27_H_29_FN_2_O_3_, MW = 448.54, [M + H]^+^ 449.20. ^1^H NMR (500 MHz, DMSO-*d*_6_) δ ppm: 7.69–7.75 (m, 4H, Ph), 7.32–7.37 (m, 2H, Ph), 7.05–7.12 (m, 3H, Ph), 6.53 (dd, *J* = 8.31, 2.00 Hz, 1H, Ph), 6.47 (t, *J* = 2.00 Hz, 1H, Ph), 6.38 (dd, *J* = 8.02, 2.29 Hz, 1H, Ph), 4.13 (t, *J* = 6.30 Hz, 2H, CH_2_-O), 3.68 (s, 3H, CH_3_), 3.31 (br. s., 4H, Pip-3,5H), 3.09 (br. s., 4H, Pip-2,6H), 3.01–3.06 (m, 2H, NCH_2_), 2.07–2.14 (m, 2H, CH_2_). ^13^C NMR (DMSO-*d*_6_): 193.63, 165.87, 164.60, 162.64, 160.78, 151.90, 134.80, 132.69, 130.33, 129.92, 116.16, 114.94, 108.79, 105.42, 102.45, 66.20, 55.47, 53.81, 51.83, 46.77, 24.61

### 3.2. Pharmacology

#### [^3^H]N^α^-Methylhistamine hH_3_R Displacement Assay

The displacement binding assay was carried out as described by Kottke et al. with slight modifications [30,31]. Frozen crude membrane preparations of HEK-293 cells stably expressing the recombinant full length hH_3_R were thawed, homogenized, and incubated for 90 min at RT (continuously shaking) with [^3^H]N^α^-Methylhistamine (2 nM) and different concentrations of the test compounds (five to eleven appropriate concentrations between 10^−11^ and 10^−4^ M, dilutions prepared robot-assisted with a Tecan Freedom EVO (Männedorf, Switzerland) or HP D300 Dispenser (Männedorf, Switzerland) in a final assay volume of 200 µL per well. Non-specific binding was determined in the presence of pitolisant (10 µM). The bound radioligand was separated from the free radioligand by filtration through GF/B filters (pretreated with 0.3% (*m/v*) polyethylenimine) using an Inotech cell harvester (Dottikin, Switzerland). Unbound radioligand was removed by three washing steps with ice-cold water. Scintillation cocktail was added, and the liquid scintillation counting was performed with a Perkin Elmer TriluxBeta counter (Perkin Elmer, Rodgau, Germany).

Scintillation data (c.p.m.) corrected for non-specific binding were analyzed using GraphPad Prism (V6.01, San Diego, CA, USA) software, using non-linear least squares/regression fit. K_i_ values were calculated from IC_50_ values according to the Cheng–Prusoff equation [35]. Statistical analyses were performed on pK_i_ values from at least three experiments, each performed, at least, in duplicates. Mean affinity values (K_i_) were transferred into nanomolar concentrations (with 95% confidence interval).

### 3.3. In Vitro Antioxidant Activity

Antioxidant properties of compounds were tested in vitro in two different assays: the 2,2-diphenyl-1-picrylhydrazyl (DPPH) assay [36,37] and the FeCl_3_ reduction activity assay (FRAP, ferric reducing antioxidant power) [37]. The tested compounds and ascorbic acid were dissolved in DMSO at the 10^−2^ mol/L concentration, and in ethanol at the concentration of 10^−4^ mol/L respectively. Moreover, 30% and 50% ascorbic acid activity cut-off for DPPH and FRAP assays respectively were set ad hoc.

#### 3.3.1. DPPH Assay

DPPH is a molecule containing a stable free radical. The reduction of DPPH (purple) in ethanol solution takes place in the presence of a hydrogen-donating antioxidant due to the formation of the non-radical form of DPPH-H (yellow). This transformation results in color change from purple to yellow, which can be measured spectrophotometrically. Thus, the decrease in absorbance is proportional to the decrease in concentration of DPPH (free radical) and discoloration of reaction mixture indicates the scavenging of free radicals by a tested antioxidant.

In order to determine antioxidant capacity, 20 μL of the compound solutions (dissolved in 96% ethanol) was mixed with 180 μL of 0.3 mM ethanolic DPPH solution. The change in the absorbance was detected at 517 nm after 30 min of incubation. Results were expressed as percentage decrease in absorbance of the tested sample, compared to the sample containing the solvent + reaction mixture (blank with maximum concentration of the DPPH radical). L-ascorbic acid was used as a reference compound. Calculations were made using MS Excel, from the proportion where the change of absorbance in the vitamin C (vit. C) group was treated as 100%, according to Equation (1):

(1) $$\%\;\mathrm{activity}\;\mathrm{of}\;\mathrm{Vit}.C=\frac{\Delta A_{b}\times 100}{\Delta A_{Vit.\;\;C}}$$

Calculation of % Vit. C activity: $\Delta A_{b}$-the change in the absorbance of the test sample and $\Delta A_{Vit.\;C}$ is change in the absorbance of reference sample, or with vit. C.

#### 3.3.2. FRAP Assay

A modified method of Benzie and Strain [38] was adopted for the FRAP assay. The stock solutions included 300 mM acetate buffer (3.1 g of C_2_H_3_NaO_2_ × 3H_2_O and 16 mL of C_2_H_4_O_2_), pH 3.6, 10 mM TPTZ (2,4,6-tripyridyl-s-triazine) solution in 40 mM HCl, and 20 mM FeCl_3_ × 6H_2_O solution. The working solution was prepared by mixing 10 parts of acetate buffer, 1 part of TPTZ solution, and 1 part of FeCl_3_ × 6H_2_O solution. A portion of 180 μL of the FRAP solution was mixed with 20 μL of the tested compound solution and incubated at room temperature for 10 min in the dark. Readings of the color product (ferrous tripyridyl triazine complex) were then taken at 593 nm against ethanol. Results are expressed as an increase in absorbance of the test sample compared to a sample containing the solvent. L-ascorbic acid was used as reference compound.

### 3.4. Molecular Modeling

For docking purposes, Schrödinger Maestro Suite (v. 11.5.011, Release 2019-01) was used [39]. Ligands were built in either their forms (protonated *N*1 piperazine nitrogen, structure charge +1) or non-ionized forms. Bioactive conformations were generated using ConfGen module [40,41] (force field: OPLS3, water environment; minimization method: Polak-Ribier Conjugate Gradient (PRCG) with maximum iterations of 2500 and a convergence threshold of 0.05; conformational search of 100 steps per rotational bond). For all compounds, the five lowest energy conformers were selected for docking studies. Possible binding pocket adaptation in the presence of certain ligands was examined using an induced fit refinement protocol [42] with **17**. In order to validate the methods used, the ligand was then re-docked with high confidence. The site was then centered on the ligand. Docking to rigid the form of the receptor was performed using the Glide module [43,44] (extra precision, flexible ligand sampling, and a maximum of five poses per conformer). Ligands were rated according to their position in the binding pocket, interactions with the binding pocket amino acids, as well as the docking score value. Ligand–receptor visualizations were generated using Schrödinger Maestro.

Theoretical pK_a_ values were calculated using the Jaguar module [45,46].

Dynamic simulations (in time of 600 ps, T = 300 K) were performed using the Nosé–Poincaré–Andersen equations of motion, forcefield AMBER10: EHT; R-Field 1:80, cutoff (8,10), and performed in MOE v. 2019.1 [47].

## 4. Conclusions

Looking for a new, active structures, is not an easy task. It even becomes harder when the reference structure is of one of the highest affinity hH_3_R ligands obtained to date; thus, increasing the possibility of ending up with lower affinity compounds. However, obtaining such compounds teaches us a lesson on structural uniqueness.

In an attempt to find new possibly dual acting H_3_R ligands, in this study we obtained a series of compounds of moderate hH_3_R affinity, ranging around *K*_i_ of 500 nM (**17**), on the one hand, and on the other hand, expressing antioxidative properties (e.g., **16**, hH_3_R *K*_i_ = 592 nM, showing 50–60% of vitamin C activity at a 10^−4^ mol/L). To our knowledge, alongside a recently published Tetratarget ligand Contilisant and its predecessor ASS234 [27], obtained herein derivatives are some of the few histamine H_3_R ligands with described antioxidative properties. From a structural point of view, it appeared that simple (cyclo)alkyl substituents at the 4-position of piperazine negatively impact the ligand’s activity at the desired target, when compared to KSK63 (Figure 1). Nonetheless, in the case of simple acyl moiety, a benzene ring separating the acyl from the basic core, through additional interactions, might force slightly higher affinity within our novel series (e.g., **10** vs. **4**), yet still on the low level. Interestingly, docking into the histamine H_3_R homology model revealed two putative binding modes, with ionic key interactions retained in both cases. The stability of such obtained complexes was demonstrated by MD simulations. This also allowed for possible explanation of KSK63 high affinity. Last, but not least, in an attempt to find possible dual acting ligands, selected compounds were tested for antioxidant properties. Among others, compound **16** (hH_3_R *K*_i_ = 592 nM) showed the strongest antioxidant properties at a 10^−4^ mol/L concentration. Compound **16** not only significantly reduced the amount of free radicals showing 50–60% of AA activity, but had also exhibited antioxidative properties in general. Despite yet unknown mechanisms and moderate hH_3_R affinity, compound **16** (QD13) constitutes a novel, potential dual acting H_3_R ligand-promising starting point towards treatment of neurologic disorders associated with increased neuronal oxidative stress.

In conclusion, although the obtained series of compounds appeared to be weak histamine H_3_ receptors ligands, they might be valuable pharmacological tools in the search for novel molecules, targeting oxidative stress-based diseases. Identification of strong histamine H_3_ receptor ligands, with antioxidative properties, will drive our further, planned studies.

## Figures and Tables

**Table 1:** Structures of compounds **4**–**18** and their in vitro human histamine H_3_ receptor (hH_3_R) affinities. Given data represent mean values within the 95% confidence interval (CI).

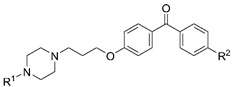			
Compound	R^1^	R^2^	hH_3_R *K*_i_ [nM] x¯ [CI 95%]
**4** QD11	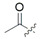	H	3653 [2024, 6594]
**5** QD15	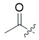	Cl	1257 [599, 2638]
**6** QD4	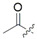	F	1100 [535, 2263]
**7** QD14	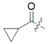	H	1334 [274, 6505]
**8** QD19	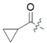	Cl	>1000
**9** QD8	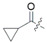	F	1669 [596, 4676]
**10** QD10	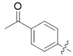	H	633 [206, 1942]
**11** QD17	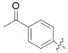	Cl	>1000
**12** QD3	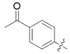	F	1008 [618, 1646]
**13** QD12	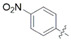	H	>1000
**14** QD20	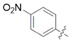	Cl	622 [211, 1832]
**15** QD6	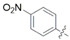	F	580 [177, 1902]
**16** QD13	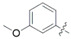	H	592 ^a^ [394, 889]
**17** QD18	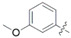	Cl	518 ^a^ [201, 1336]
**18** QD9	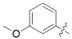	F	715 [352, 1453]

^a^ Values were obtained using 0.025% HCl in binding buffer. Values in square brackets indicate the confidence interval of 95%. *K*_i_-inhibitory constant.

**Table 2:** Absorbance changes in the 2,2-diphenyl-1-picrylhydrazyl (DPPH) and ferric reducing antioxidant power (FRAP) assays. The highest obtained values for the tested compounds were marked in **bold.**

Compound	DPPH Assay (λ = 517 nm)	FRAP Assay (λ = 593 nm)
	Absorbance Decrease in 10^−4^ mol/L Concentration [%] Mean ± SEM	Absorbance Increase in 10^−4^ mol/L Concentration [%] Mean ± SEM
Ascorbic acid	20.52 ± 0.51	344.20 ± 4.65
**6**	8.82 ± 0.12	0.79 ± 0.39
**7**	6.23 ± 0.53	1.43 ± 0.32
**8**	6.79 ± 0.51	0.48 ± 0.48
**9**	7.70 ± 0.41	1.75 ± 0.22
**10**	2.44 ± 0.97	3.66 ± 0.59
**11**	3.33 ± 0.45	3.34 ± 0.22
**12**	4.42 ± 0.33	2.70 ± 1.15
**14**	6.31 ± 1.14	0.22 ± 0.16
**16**	**12.37 ± 1.31**	**32.91 ± 0.81**
**17**	4.88 ± 0.18	**40.86 ± 0.98**
**18**	6.35 ± 0.55	**29.73 ± 0.39**

## Data Availability

The data presented in this study are available on request from the corresponding author.

## Supplementary Materials

The following are available online, The Supplementary Material contain Table S1: Calculated pKa values for piperazine *N1* and *N*4 nitrogen, Table S2: Putative binding poses for non-protonated conformers, Table S3: Putative binding poses for protonated conformers, Table S4: Molecular dynamics frames alignment for selected ligands and Figure S1: Antioxidant activity of the tested compounds in DPPH and FRAP assay.
