# Supplementary material for: Discovery of Potential, Dual-Active Histamine H3 Receptor Ligands with Combined Antioxidant Properties
Source: Molecules. 2021 Apr 15;26(8):2300. doi: 10.3390/molecules26082300 (PMC8071534; doi:10.3390/molecules26082300)
Supplement: Supplementary file 1 [file molecules-26-02300-s001.zip › molecules-1161308-supplementary.pdf]

molecules

Article – Supplementary material

H-bond acceptor-substituted piperazine derivatives as potential histamine H_3 receptor ligands with antioxidant properties

Kamil J. Kuder[1*], Magdalena Kotańska[2], Katarzyna Szczepańska[1], Kamil Mika[2], David Reiner[3], Holger Stark[3], Katarzyna Kieć-Kononowicz[1]

1. pKa calculations (Jaguar) 1
2. Antioxidant activity of the tested compounds 2
3. Molecular modeling 3

1. pKa calculations (Jaguar)

Table S1 Calculated pKa values for piperazine *N1* and *N4* nitrogen

Compound	*N*1 p*K*a	*N*4 p*K*a	Compound	*N*1 p*K*a	*N*4 p*K*a
**4**	6.5	-6.0	**13**	6.6	-4.2
**5**	6.5	-6.0	**14**	6.6	-4.2
**6**	6.5	-6.0	**15**	6.6	-4.2
**7**	6.7	-5.2	**16**	6.4	-0.5
**8**	6.7	-5.2	**17**	6.4	-0.5
**9**	6.7	-5.2	**18**	6.4	-0.5
**10**	5.4	-6.1	KSK63	6.8	-1.1
**11**	5.4	-6.1			
**12**	5.4	-6.1			

2. Antioxidant activity of the tested compounds

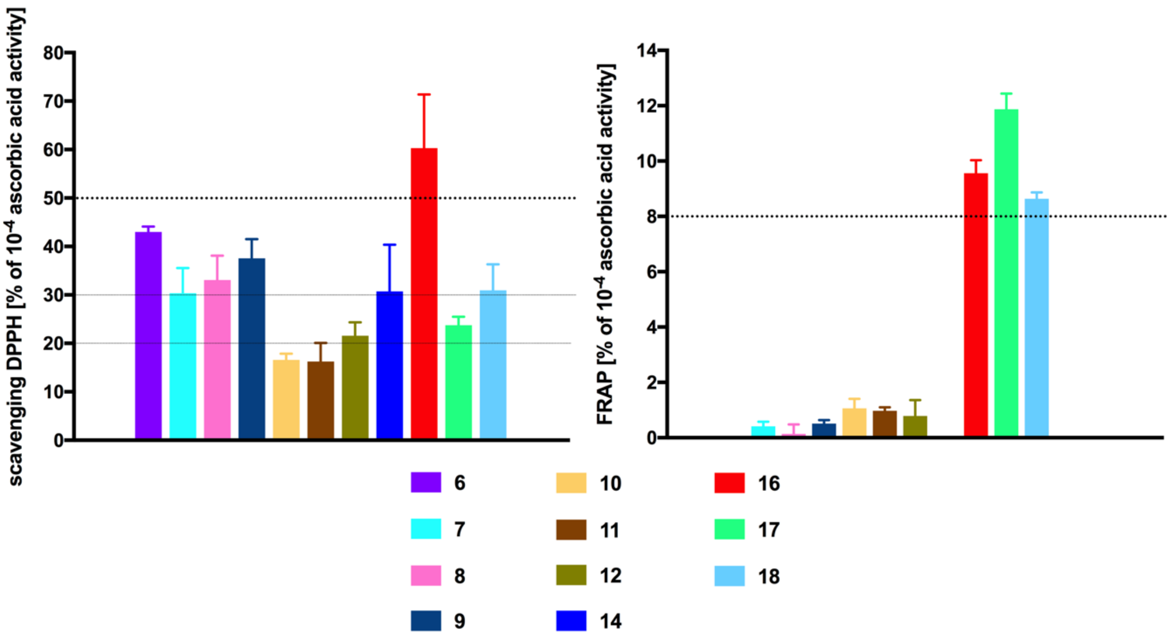

Figure S1 Antioxidant activity of the tested compounds inDPPH and FRAP assay

3. Molecular modeling

Table S2 Putative binding poses for non-protonated conformers

4 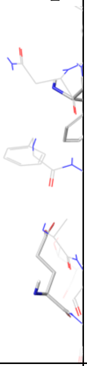	5 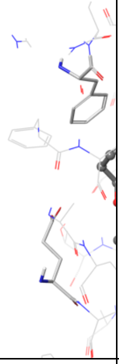	6 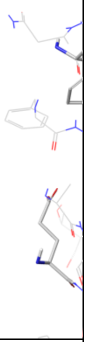
7 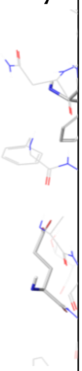	8 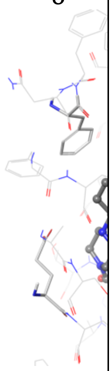	9 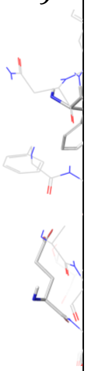
10 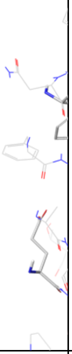	11 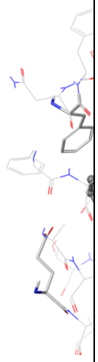	12 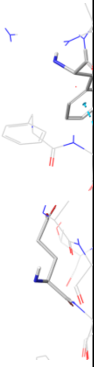
13 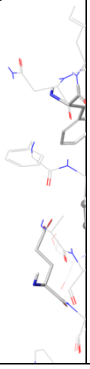	14 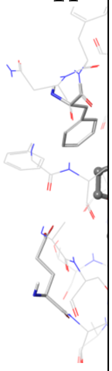	15 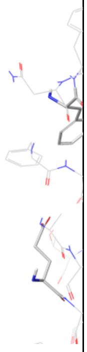

**16** 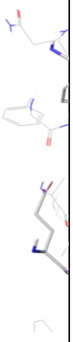	**17** 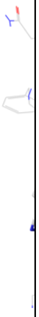	**18** 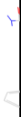

Table S3 Putative binding poses for protonated conformers

**4** 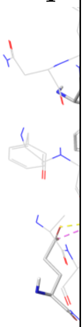	**5** 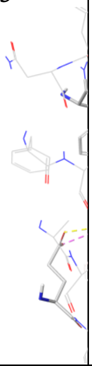	**6** 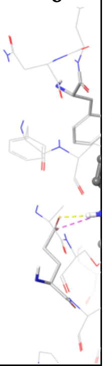
**7** 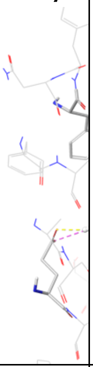	**8** 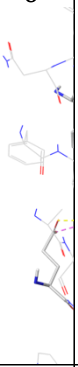	**9** 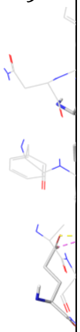
**10** 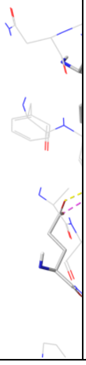	**11** 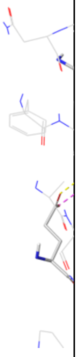	**12** 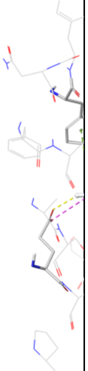

**13** 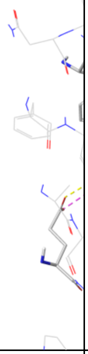	**14** 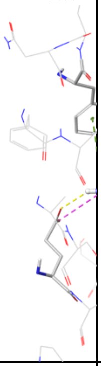	**15** 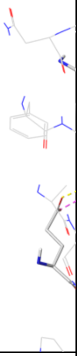
**1**<br>**6** 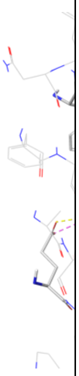	**17** 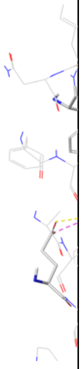	**18**<br>"stand-<br>ard" 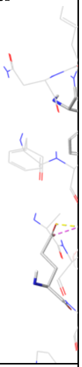
		**18**<br>"up-<br>side-<br>down" 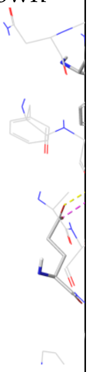

Table S4 Molecular dynamics frames alignment for selected ligands. 0ps–grey, 100ps–green, 200ps–teal, 300ps–green, 400ps–violet, 500ps–orange, 600ps–yellow

**10** 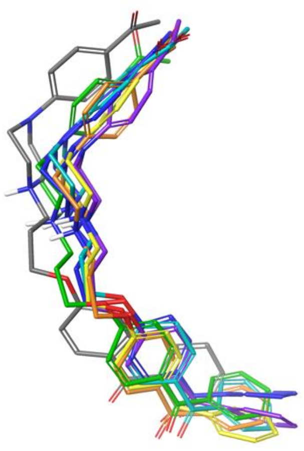	**14** 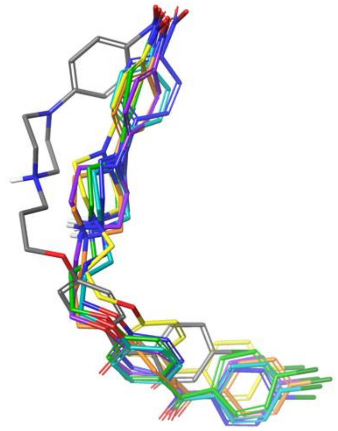
**15** 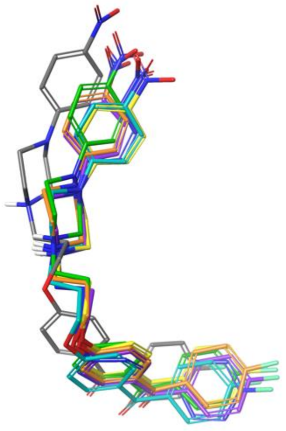	**16** 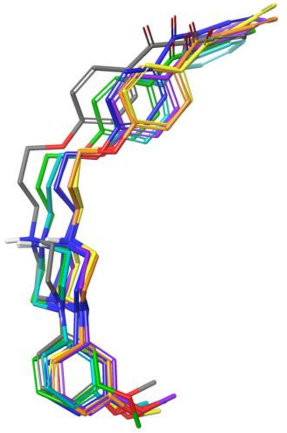
**17** 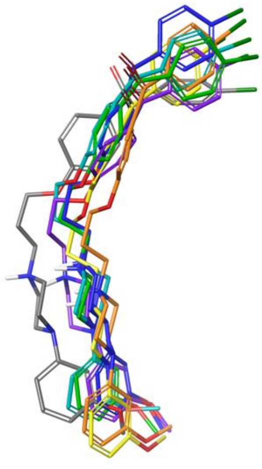	**18** “standard” 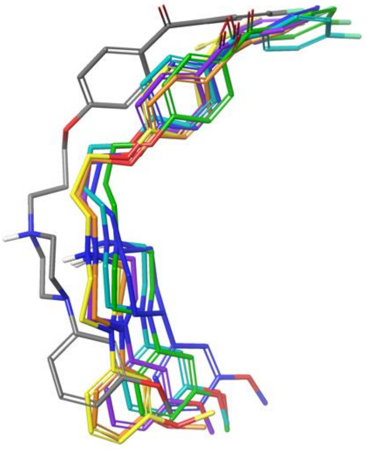

	**18** "upside-down" 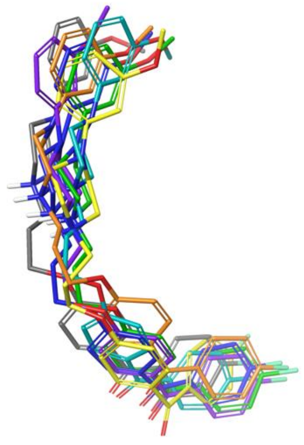
